# Vascular tumors of the external auditory canal: three case reports and a review of the literature

**DOI:** 10.1186/s40064-015-1113-5

**Published:** 2015-07-01

**Authors:** Keisuke Yamamoto, Noriko Ogasawara, Nobuhiko Seki, Nobuhiro Konno, Hiroshi Tsubota, Tetsuo Himi

**Affiliations:** Department of Otorhinolaryngology, Sapporo Medical University School of Medicine, Minami 1-jo Nishi 16-chome, Chuo-ku, Sapporo, Hokkaido 060-0061 Japan; Department of Otolaryngology, Hakodate Municipal Hospital, 10-1, Minato-cho 1-chome, Hakodate, Hokkaido 041-8680 Japan

**Keywords:** Vascular malformations, Benign external auditory canal tumor, Hemangioma, Vascular anomalies

## Abstract

**Background:**

Benign vascular tumors are frequently found in the head and neck, however, such tumors of the external auditory canal are extremely rare. We report three cases of benign vascular tumors limited to the external auditory canal.

**Case description:**

A 50-year-old woman was diagnosed during an episode of ear fullness and hearing loss. A 10-year-old boy consulted our department about an episode of recurrent otorrhagia. A 20-year-old man found a bulge of his external auditory canal by chance. Complete surgical resection was performed for the first patient. The second patient underwent electro-coagulation of the lesion. In the third patient, to exclude the possibility of a malignant tumor, a biopsy was performed under local anesthesia. Histopathological analysis demonstrated the characteristic of vascular tumors. The lesion showed remarkable reduction during his treatment with antibiotics and cleaning. He remains under careful observation.

**Discussion and evaluation:**

In diagnosis, there is sometimes confusion between vascular tumors and malformations. Generally, vascular malformations can be differentiated from vascular tumors since they are present at birth and are generally stable.

**Conclusion:**

Decision making about treatment of benign vascular tumors is sometimes confusing because of the difficulty in diagnosis. We performed biopsy for only one of our three cases because we regard that informal biopsy should not be conducted for lesions with difficult hemostic conditions and locations.

**Electronic supplementary material:**

The online version of this article (doi:10.1186/s40064-015-1113-5) contains supplementary material, which is available to authorized users.

## Background

In 1996, the International Society for the Study of Vascular Anomalies (ISSVA) advocated that the ISSVA classification (Mulliken and Glowacki 1982) in which “hemangioma” was divided into two main categories, such as vascular tumors and vascular malformations. In vascular tumors, endothelial cells grow rapidly (proliferating phase), and subsequently an involution phase follows for several years. Infant hemangioma is the most common, and pyogenic granuloma is also classified as a vascular tumor. On the other hand, vascular malformations are caused by abnormal origination from peripheral vascular formation during embryogenesis. Generally such malformations grow slowly and do not disappear naturally (Luca et al. 2007). Vascular malformations are classified into four types: capillary malformation, venous malformation, lymphatic malformation and arteriovenous malformation. Although vascular lesions are frequently found in the head and neck, they are extremely rare in the external auditory canal. We report here three cases of vascular tumors localized in the external auditory canal.

## Case description

### Case 1

**Figure 1:**
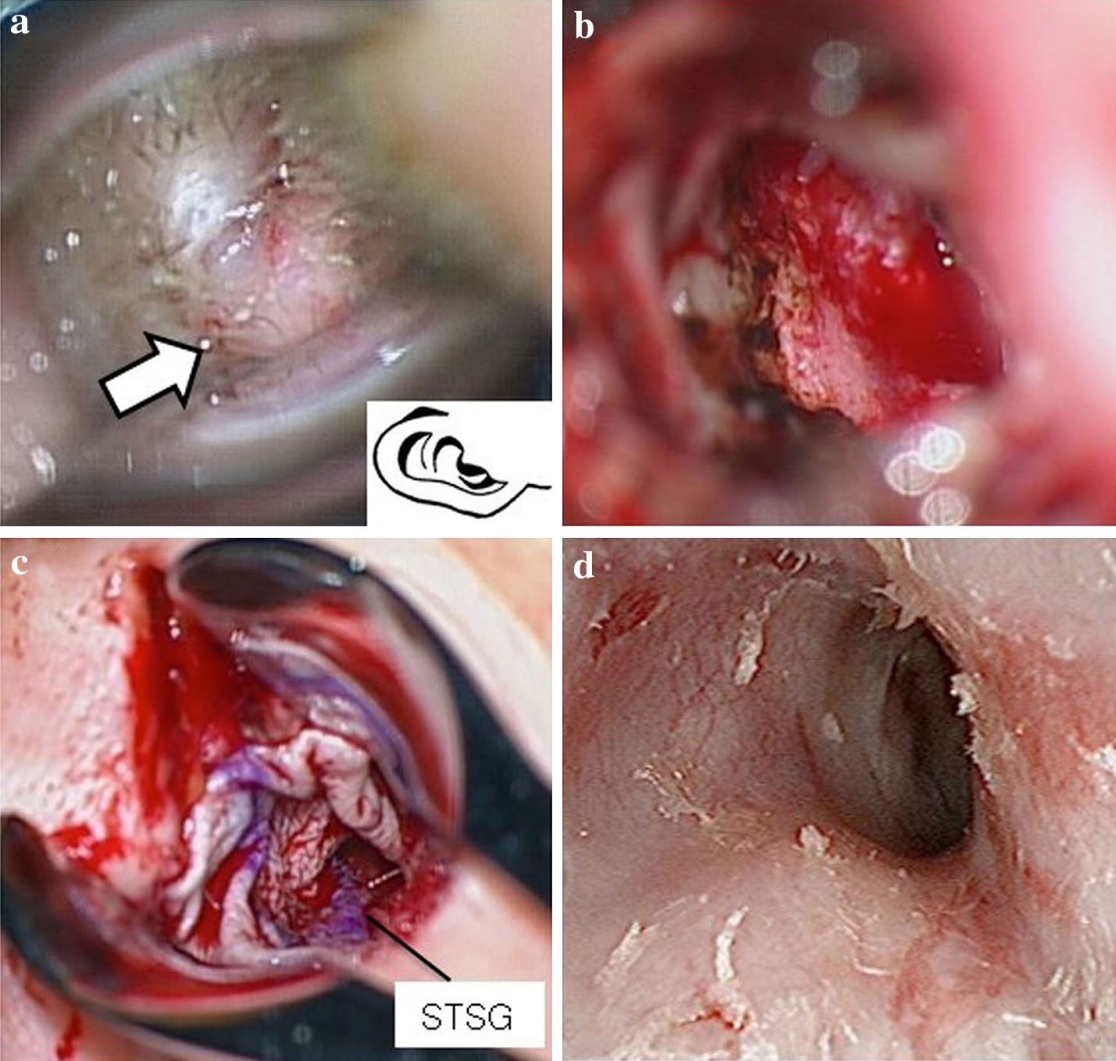
Case 1: **a** Right otoscopic view. A soft mass (*arrow*) totally fills the external auricular canals hiding the entire tympanic membrane. **b** Operative findings. By transcanal and post auricular approaches, the skin with the tumor was fully removed from the external auditory canal leaving part of the anterior wall. **c** 2 × 2 cm split thickness skin graft excised from the right thigh was epidermizationed into the right extra auricular canal from its entrance to the outside of the eardrum ring. **d** The otoscopic finding 8 months after the operation. No recurrence of the vascular lesion was seen in the external auricular canal or tympanic membrane.

A 50-year-old woman was referred to our hospital for investigation of a mass in the right external auditory canal. She had suffered from hearing loss and ear fullness in her right ear for some months. She had no significant past medical history. On physical examination, right otoscopy revealed a soft mass totally filling the external auditory canal and hiding the entire tympanic membrane (Figure 1). Right threshold of hearing (averaged over 0.5, 1, 2 and 4 kHz) was 51.25 dB with conductive hearing loss. A tympanogram showed type B in her right ear.

**Figure 2:**
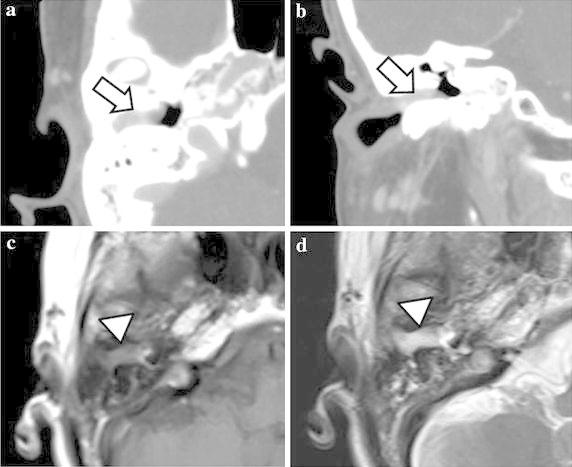
Case 1: Enhanced CT scan of temporal bone, axial view **a** and coronal view **b** demonstrates a soft-tissue mass (*arrow*) lateral to the tympanic membrane that fills the right external auditory canal without involvement of the tympanum. There is no evidence of bone erosion, and the tympanic membrane, middle ear, and ossicles appear normal. Magnetic resonance imaging (MRI) of the skull base shows an external auditory canal mass (*arrowhead*) that is slightly hypointense in a T1-weighted image (**c**), and hyperintense in T2-weighted images (**d**).

An enhanced temporal bone computed tomography (CT) scan demonstrated a soft-tissue mass lateral to the tympanic membrane that filled the right external auditory canal without involvement of the tympanum. There was no evidence of bone erosion. The tympanic membrane and the ossicles appeared normal. Magnetic resonance imaging (MRI) of the temporal bone demonstrated a slightly hypointense external auditory canal mass in T1-weighted images, and hyperintensity in T2-weighted images (Figure 2). This finding was considered to be a vascular lesion. There were no feeding vessels to a mass found by magnetic resonance angiography (MRA).

The lesion was removed using a transcanal and retroauricular approaches under general anesthesia. The base of the lesion was found to be in the posterior wall. The lesion was close to the annulus tympanicus, but did not adhere to the tympanic membrane. The tumor and skin were fully removed from the external auditory canal leaving a part of the anterior wall. We could not identify the feeding vessels. Total bleeding during the operation was 30 ml. Bleeding was easily controlled by monopolar and bipolar coagulation. We transplanted a skin graft excised from her right thigh to the right external auditory canal (Figure 1). Ten days after surgery, the skin was fully engrafted. Eleven days after surgery, she was discharged from our hospital.

**Figure 3:**
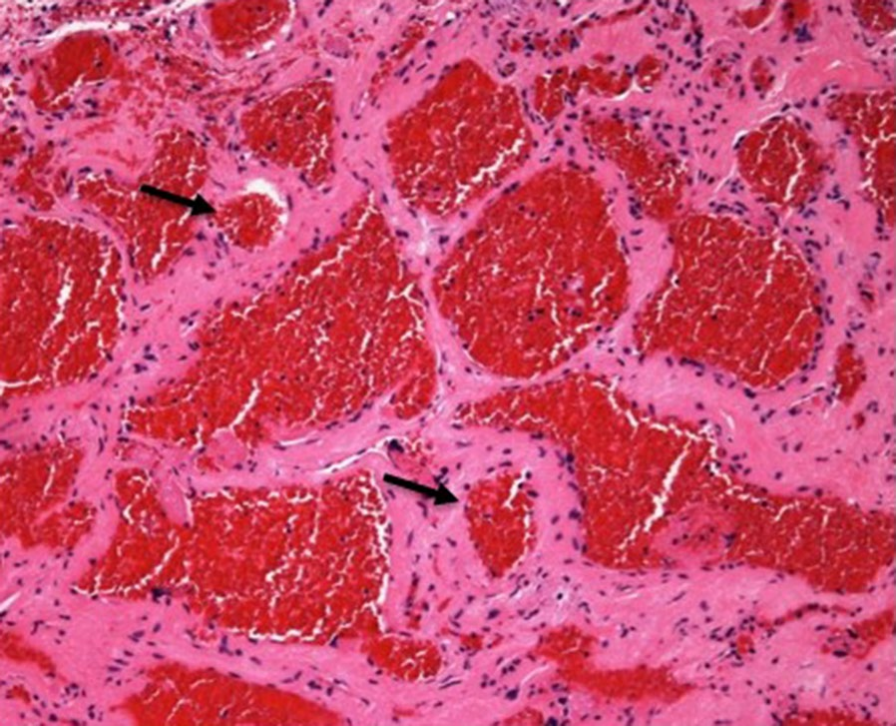
Case 1: **a** Pathogenic examination reveals that capillary vessels (*arrow*) consisting of a monolayer of endothelial cells increasing in the epidermis. This appearance is characteristic of vascular tumors (haematoxylin and eosin, original magnification ×200).

Histopathological analysis revealed capillary vessels consisting of a monolayer of endothelial cells increasing in the epidermis (Figure 3). This appearance was characteristic of vascular tumors. The postoperative course was uneventful. An audiogram 8 months after her operation showed notable improvement of the air-bone gap and 33.75 dB of right threshold of hearing (averaged over 0.5, 1, 2 and 4 kHz)..

### Case 2

A 10-year-old boy presented with recurrent purulent ear discharge from his left of external auditory canal. On physical examination, three red lesions the 1–4 mm in diameter were observed at the entrance of the left external auditory canal. No other skin lesion was observed on his head, neck, trunk or limbs.

The tympanic membrane did not show abnormality. A pure tone audiogram and tympanograms exhibited the normal range. The lesions were so small and they were not recognized in contrast enhanced CT and MRI with gadolinium administration. After 5 months of follow-up, he and his mother hoped for radical treatment to suppress recurrent purulent ear discharge. We informed them about various possible treatments, including sclerotherapy or a local flap. Since the tumor was small, we assessed the possibility of external auditory canal stenosis caused by coagulation to be low. We coagulated his lesions using a bipolar electrical scalpel under local anesthesia. This patient remains free of recurrence at 6 months post-coagulation.

### Case 3

**Figure 4:**
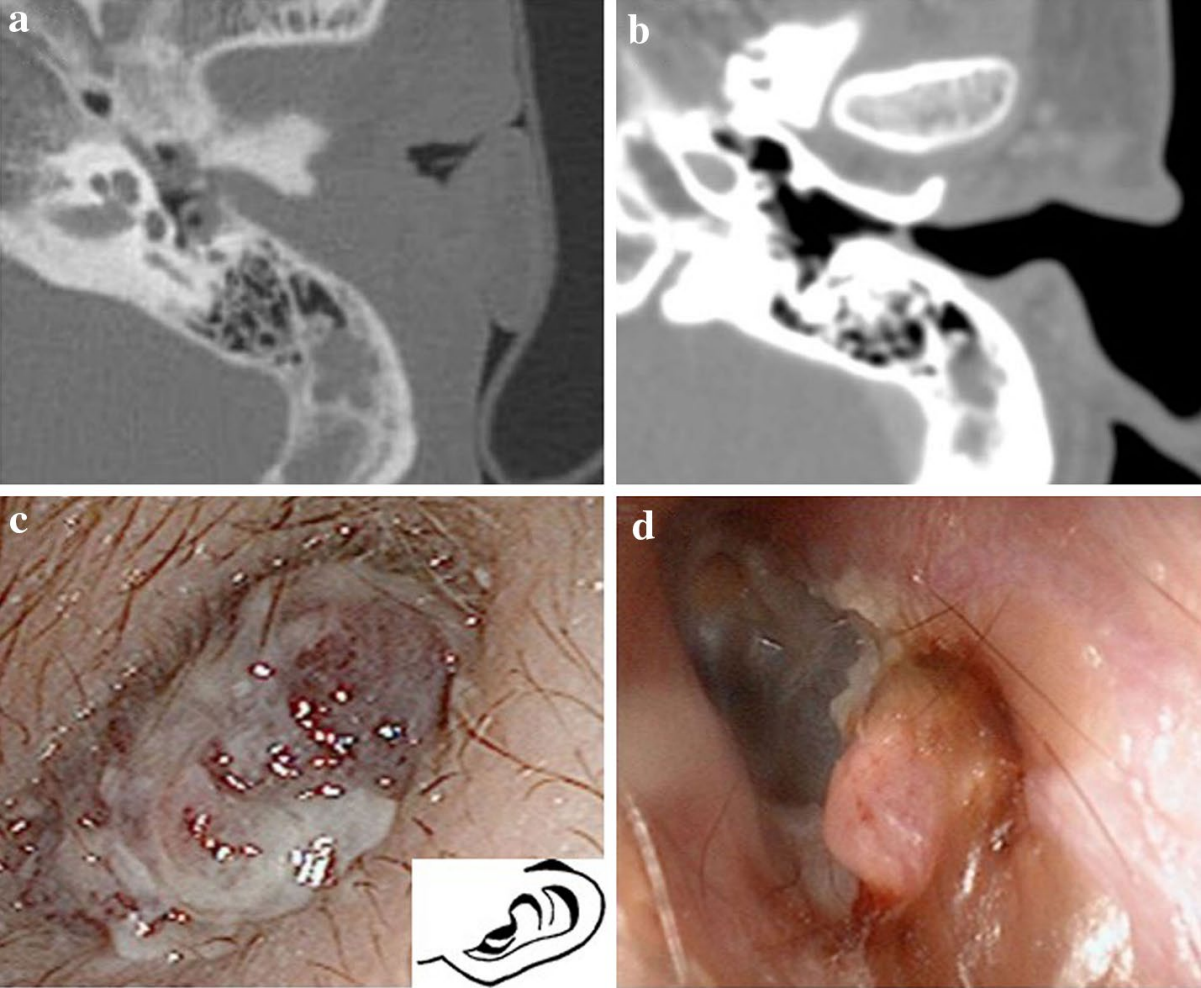
Case 3: **a** Soft-tissue mass filling the left auditory canal without involvement of the bony canal when he was treated for otitis media 4 months before our medical examination. **b** An enhanced CT scan of the temporal bone reveals an isolated enhanced soft tissue mass in the posterior-inferior wall. **c** Left otoscopy reveals red granulation tissue, and the tympanic membrane cannot be recognized. In later otoscopy **d**, the mass was markedly smaller.

**Figure 5:**
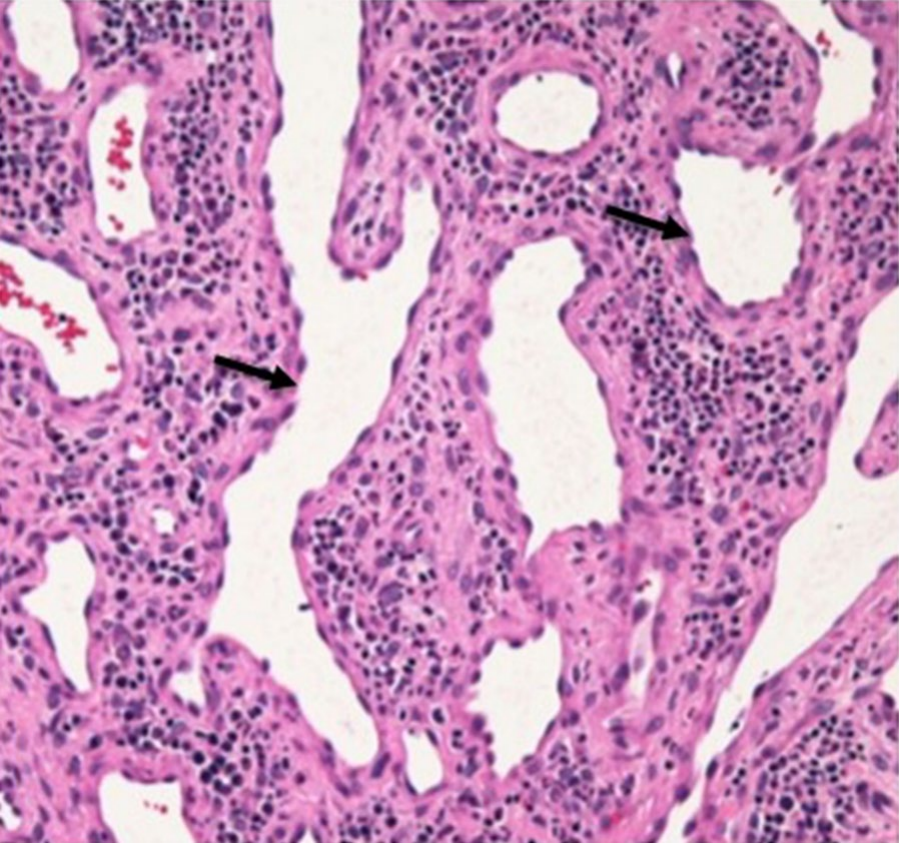
Case 3: Microscopic examination of the tissue demonstrated expanded vessels (*arrow*) increased with inflammatory cells. The appearance is characteristic of vascular tumors (haematoxylin and eosin, original magnification ×200).

A 20-year-old man with cerebral palsy presented with a lesion in his left external auditory canal. Once 4 months earlier he had been treated for otitis media with ofloxacin otic solution for a week by a pediatrician. A CT scan revealed soft-tissue lesions that filled the external auditory canal without erosion of the bony canal. Otoscopy revealed red granulation tissue and his left tympanic membrane could not be seen (Figure 4). To exclude the possibility of a malignant tumor, a biopsy was performed under local anesthesia and careful preparations for hemostasis. Histopathological analysis demonstrated expanded vessels increased with inflammatory cells. The appearance was characteristic of vascular tumors (Figure 5). We administered antibiotics for few days and cleaned his extra auditory canal regularly. Four months after his first visit, otoscopy findings showed marked reduction of the lesion. No sign of relapse has been detected during 2 years after the biopsy.

## Discussion and evaluation

External auditory canal tumors are extremely rare. Mangham et al. (1981) reported that only ten vascular tumors (vascular malformation, hemangioma) were observed among 1,430 cases of temporal bone tumors (0.7%), and they usually occurred in the internal auditory canal or along the course of the facial nerve. Twelve cases of external auditory canal vascular lesions excluding the tympanic membrane have been reported to date (Luca et al. 2007; Hawke and van Nostrand 1987; Krueger and Porto 1988; Limb et al. 2002; Reeck et al. 2002; Yang et al. 2006; Verret et al. 2007; Covelli et al. 2008; Rutherford and Leonard 2010; Martines et al. 2012; Shu et al. 2013) (Additional file 1: Table S1). The patients ranged from 10 to 67 years old, and 11 of them were male. Eight cases were in the left ear and in one case it was not reported which side was affected. The most common chief complaint was hearing loss (8 cases), followed by ear fullness and otorrhagia (5 cases).

First of all, malignant disease must be excluded by histopathology. However biopsy without adequate preparations may lead to severe bleeding. Hawke and van Nostrand (1987) reported a case that required electric coagulation under general anesthesia because manipulation caused significant arterial bleeding that did not respond to adrenalin-soaked gauze.

Computed tomography (CT), more precisely enhanced CT of the temporal bone, and/or magnetic resonance imaging (MRI) are effective to evaluate the extent of the tumor, bone erosions and middle ear involvement. Typical MRI features of vascular lesions are a hypointense or isointense lesion on T1-weighted images, and hyperintensity on T2-weighted images, with intense enhancement on gadolinium administration (Luca et al. 2007). In Case 1, both MRI and enhanced CT demonstrated that the tumor was limited to the external auditory canal and we could remove it by surgical resection. It is considered that feeder vessels must be identified using angiography, or MRA examination before radical surgery (Limb et al. 2002).

It is hard to distinguish Case 2 between vascular tumors and vascular malformations without histopathological examination. Increase of the blood vessels is the findings of vascular tumors in Case 2 and Case 3. It seems that vascular lesions frequently include pyogenic granuloma such as that in Case 3, because histologically the expansion of capillaries was showed and the size of the tumor was decreased by local cleaning and antibiotics administration. Though pyogenic granuloma is benign, it frequently shows rapid enlargement. Pyogenic granuloma in the external auditory canal is also extremely rare. To date, only three cases have been reported in the English literature (Casler et al. 1989; Courtney et al. 2003; Hsu et al. 2008). It is said that vascular tumors exists first and then develops into pyogenic granuloma as a results of infection, injury and chronic stimulation. In Case 3, it is likely that an infection enlarged an original vascular tumor because antibiotic administration and local cleaning markedly reduced the size of the lesion.

Vascular lesions in the external auditory canal might be treated by various manners such as radical resection, laser solidification, electrocoagulation, embolus therapy, sclerotherapy or radiotherapy. In 10 of the 12 reported cases, radical operation was performed for the lesion, whereas the others kept under observation. Zinis reported that there was no need for treatment unless the lesion showed especially rapid growth (Luca et al. 2007). However, we have shown that conductive hearing loss by vascular tumors localized in the external auditory canal can be treated. Besides, otorrhagia due to small vascular malformation at an entrance of an external auditory canal was easily coagulated using a bipolar electrical scalpel under local anesthesia.

## Conclusion

We believe that in cases in which malignancy cannot be excluded and antibiotic administration has no effect, we must perform biopsy of the lesion after taking sufficient precautions against bleeding. We reported three cases of vascular tumors limited to the external auditory canal. Such lesions are extremely rare. To date, only 12 other cases of the vascular lesion limited to external auditory canal have been reported in the English literature. Radical resection of the vascular tumors was valuable in improvement of the conductive hearing loss, and electro-coagulation was useful for small vascular lesions at an entrance of an external auditory canal. On the other hand, there is no need to resect some vascular lesions by using antibiotics without some symptoms or malignant disease.

## Additional file

Additional file 1: 
**Table** **S1.** Hemangioma limited to the external auricular canal reported in the English literature.

## Abbreviations

CT: computed tomography
MRI: magnetic resonance imaging
MRA: magnetic resonance angiography
ISSVA: the International Society for the Study of Vascular Anomalies
