# Supplementary material for: Vascular tumors of the external auditory canal: three case reports and a review of the literature
Source: Springerplus. 2015 Jul 1;4:307. doi: 10.1186/s40064-015-1113-5 (PMC4486653; doi:10.1186/s40064-015-1113-5)
Supplement: Additional file 1: — Table S1. Hemangioma limited to the external auricular canal reported in the English literature. [file 40064_2015_1113_MOESM1_ESM.pdf]

Table 1 Hemangioma limited to the external auricular canal reported in the English literature.

Authors	Year	age/sex	side	Base implant	Symptoms	pathology	management
Hawke et al [4].	1987	55/M	Right	Inferior canal wall	otorrhea, otorrhagia	Cavernous	Endaural excision
Krueger et al [5].	1988	50/M	Right	Postero-superior canal wall	ear fullness	Capillary	Endaural excision
Limb et al [6].	2002	67/F	Left	Postero-superior canal wall	hearing loss, tinnitus, ear fullness	Cavernous	Endaural excision, epidermization
Reek et al [7].	2002	53/M	Left	Postero-inferior canal wall	hearing loss, tinnitus	Cavernous	Endaural excision, skin flaps
Yang et al [8].	2006	72/F	Left	Superior canal wall	no symptoms	Cavernous	Endaural excision
Verret et al [9].	2007	31/M	Right	not shown	hearing loss, discomfort	not shown	post-auricular excision, epidermization
Luca et al [2].	2007	74/M	Left	Posterior canal wall	otorrhagia, otalgia	not examined	observation
Luca et al [2].	2007	53/M	not shown	Antero-inferior canal wall	hearing loss	not examined	observation
Covelli et al [10].	2007	45/M	Left	Antero-superior canal wall	hearing loss	Cavernous	Endaural excision, skin flaps
Ruthford et al [11].	2009	62/M	Left	Antero-inferior canal wall	otorrhagia, ear fullness	Cavernous	1.post-auricular excision, 2.Trans-canal excision
Martines et al [12].	2012	59/M	Right	Postero-inferior canal wall	hearing loss, tinnitus, otorrhagia, ear fullness	Cavernous	Endaural excision
Shu et al [13].	2013	57/F	Right	Superior canal wall	otalgia	Cavernous	post-auricular excision
Yamamoto et al.	2015	50s/F	Right	posterior wall	ear fullness, hearing loss	Capillary	Endaural excision＋post-auricular excision, epidermization
Yamamoto et al.	2015	10s/M	Left	Entrance of EAC	otorrhagia, pruritus	not examined	electric coagulation
Yamamoto et al.	2015	20s/M	Left	Posterior canal wall	tumor	Capillary	observation
